# Accuracy of Single-Step versus 2-Step Double-Mix Impression Technique

**DOI:** 10.5402/2011/341546

**Published:** 2011-07-25

**Authors:** Eduardo Batista Franco, Leonardo Fernandes da Cunha, Francyle Simões Herrera, Ana Raquel Benetti

**Affiliations:** Department of Operative Dentistry, Endodontics, and Dental Materials, Bauru School of Dentistry, University of São Paulo, Al. Octávio Pinheiro Brisolla, 9-75 Vila Universitária, 17012-901 Bauru, SP, Brazil

## Abstract

*Objective*. To investigate the accuracy of dies obtained from single-step and 2-step double-mix impressions. *Material and Methods*. Impressions (*n* = 10) of a stainless steel die simulating a complete crown preparation were performed using a polyether (Impregum Soft Heavy and Light body) and a vinyl polysiloxane (Perfectim Blue Velvet and Flexi-Velvet) in two consistencies, in one or two (without relief) steps. Accuracy of the stone dies was accessed at a measuring microscope, using a metallic crown with perfect fit to the reference crown preparation. Data were submitted to 2-way ANOVA and Tukey test (*α* = 0.05). *Results*. The single-step technique resulted in slightly larger dies, while the 2-step technique without relief produced significantly smaller dies, when compared to the original stainless steel die. Stone dies obtained from 2-step polyether impressions were significantly smaller when compared to dies obtained from 2-step vinyl polysiloxane impressions (Impregum 2-step: −290.94 ± 71.64 *μ*m; Perfectim 2-step: −201.86 ± 28.58 *μ*m). No significant differences were observed in dies obtained from either polyether or vinyl polysiloxane with the single-step technique (Impregum single-step: 63.52 ± 16.60 *μ*m; Perfectim single-step: 79.40 ± 14.11 *μ*m). *Conclusion*. Higher discrepancies were detected for the 2-step impression technique without relief for the investigated materials.

## 1. Introduction

The advance in materials and development of techniques has been essential to improve the accuracy of impressions. Although vinyl polysiloxanes and polyethers are recognized for their dimensional stability [1, 2], the impression technique is a critical factor affecting this accuracy [2–10]. 

On behalf of the several impression protocols suggested, the double-mix techniques, in which two materials of different viscosity are used together [5, 7–9, 11, 12], have been preferred especially when polyether and vinyl polysiloxane materials are adopted. Single-step or 2-step procedures may be performed with putty and light body, putty and medium body, or heavy body and light body [5, 7–9, 11]. 

Single-step technique, in which both materials polymerize simultaneously, reduces chairside time and saves impression material. Although time is a limiting factor since the professional has to accommodate both low- and high-consistency materials simultaneously before setting occurs, this technique yields accurate impressions independently of the curing kinetics of the syringed material alone [12]. According to the literature, the single-step technique with vinyl polysiloxanes or polyethers leads to very accurate impressions [5–7, 11, 13].

In the 2-step technique, a high-viscosity material is used for a preliminary impression, while the final impression is performed with a lower-viscosity material. Even though the 2-step technique has been widely adopted and can offer good accuracy [3, 7, 8], some problems may be experimented with this technique, such as dimensional alterations [5, 12, 13], extra chairside time, and extra material needed [5]. 

Among the 2-step procedures, the introduction of the hydraulic and hydrophobic impression technique [14] has given a new perspective for impression taking. It is a 2-step dual-arch technique in which a preliminary impression made with a high-consistency material is relined with a lower-consistency material, both especially developed for the executing of this technique. According to this technique, the high-hardness property of the high-consistency vinyl polysiloxane is supposed to generate a hydraulic pressure that propels the low-consistency impression material into the sulcus and all the internal aspects of the preparation, eliminating the need for packing retraction cord or using die spacers. 

Despite the operational simplicity of the hydraulic and hydrophobic technique, the accuracy of this impression technique requires careful investigation. Three-dimensional measurements have already detected that dual-arch impressions result in less accurate stone dies when compared to custom-tray impressions performed with either polyether or vinyl polysiloxane [4]. Therefore, it is interesting to check to what extent the material itself may compensate the stresses generated during the relining of the high-consistency material with the low-consistency vinyl polysiloxane. Idris et al. [11], Nissan et al. [7, 8], and Caputi and Varvara [3] demonstrated that the 2-step putty-wash technique performed with vinyl polysiloxane produced very accurate stone dies when a 2 mm relief was left in the preliminary impression. Hung et al. [5] observed that the use of a plastic spacer on the master model during the preliminary impression resulted in 2-step impressions as accurate as the single-step technique. The 2-step hydraulic and hydrophobic technique, however, advises no relief of the preliminary impression. 

Therefore, the aim of this study was to investigate the accuracy of dies obtained from single-step and 2-step hydraulic and hydrophobic techniques performed with distinct impression materials. The null hypotheses to be tested were that there would be no significant differences in the accuracy of dies obtained from different materials or impression techniques.

## 2. Material and Methods

**Figure 1:**
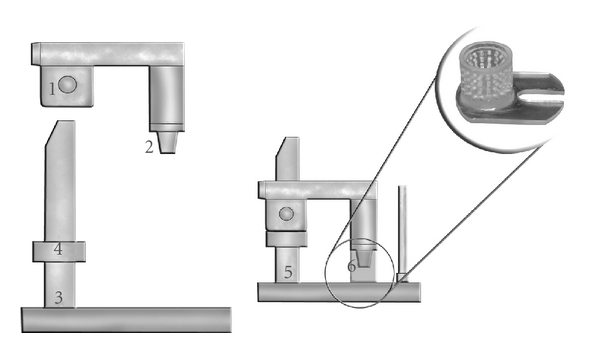
Schematic drawing of apparatus used for impression taking (1) upper device that slides on vertical axis of base; (2) steel die; (3) base; (4) stop on vertical axis; (5) fit of upper device and base; (6) perforated acrylic tray which was fixed at base of impression device.

The materials used were a polyether (Impregum Soft; 3M ESPE, Seefeld, Germany) and a vinyl polysiloxane (Perfectim; J. Morita USA Inc., Irvine, Calif, USA). In order to obtain the impressions, a stainless steel die simulating a full crown preparation (8 mm cervical diameter, 6 mm occlusal diameter, and 8 mm height) was fixed in an impression device (Figure 1) [15]. A mark in the occlusal surface of the steel die guided the adaptation of a metallic crown with an opening on its occlusal surface, which was leveled at zero to the steel die (perfect fit).

Perforated acrylic cylindrical trays (12 mm diameter) were attached to the lower part of the device. The steel die, attached to the upper part of the stand, was centralized in the acrylic tray to obtain impressions with a material thickness of approximately 2 mm. This was measured from the distance of the tray to the gingival preparation margin of the steel die. A distance of 2 mm between the top surface of the preparation and the depth of the tray was maintained.

The impression materials were mixed according to the manufacturer's instructions at controlled temperature (22 ± 1°C) and humidity (55 ± 5%). Proportion of the materials was established by weight in a precision scale. Since the double-mix techniques were investigated, high-consistency (Impregum Soft Heavy Body (ISH), 3M ESPE; Perfectim Blue Velvet (PBV), J. Morita) and low-consistency (Impregum Soft Light Body (ISL), 3M ESPE; Perfectim Flexi-Velvet (PFV), J. Morita) materials were adopted for both polyether and vinyl polysiloxane. Mixing was accomplished on a glass plate with a metallic spatula until obtaining a homogeneous mixture within 30 seconds. 

**Figure 2:**
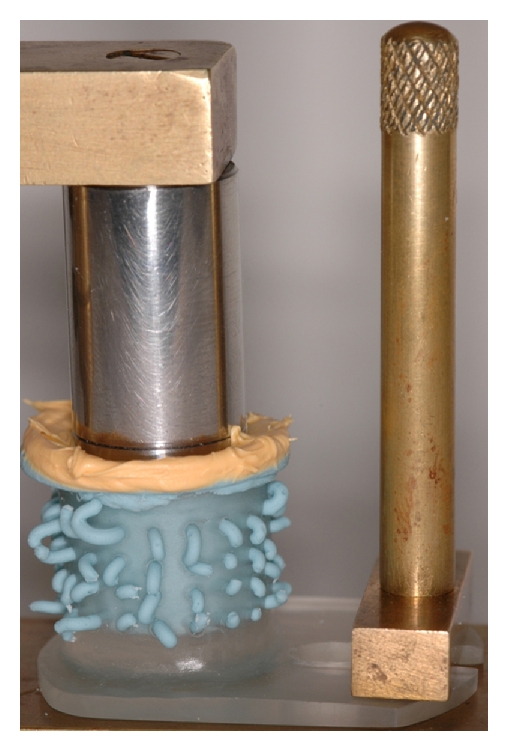
Impression of the stainless steel die using a perforated acrylic cylindrical tray attached to the impression apparatus.

Single-step and 2-step techniques were performed to obtain impression with both materials (Figure 2). For the single-step technique, the high-consistency material (ISH or PBV) was inserted in the tray, while the low-consistency material (ISL or PFV) was simultaneously spread on the steel die. For the 2-step technique, a preliminary impression was taken with the high-consistency material (ISH or PBV) and relined with the low-viscosity product (ISL or PFV). No die spacers or relief of the preliminary impression were carried out, in order to simulate the hydraulic and hydrophobic technique.

The impressions were separated from the preparation with single axial movement after 10 minutes and stored at room temperature for 2 hours to allow elastic recovery of the elastomeric materials and release of hydrogen from the vinyl polysiloxane. Improved die stone type IV (Durone, Dentsply, Petrópolis, Rio de Janeiro, Brazil) in a 0.19 water to powder ratio was mixed and poured into the impression under mechanical vibration. A dam of adhesive tape was placed around the tray to allow the construction of a cylindrical base of 8 mm height. Ten dies were produced for each experimental condition (materials and techniques).

After 2 hours, the stone die was separated from the impression and transferred to a stand in a measuring microscope (Depth Measuring Microscope Carl Zeiss 4987926, Zeiss, Jena, Germany). The fit of the metallic crown in the dies determined the dimensional accuracy of the samples when compared with the steel die. The opening on the occlusal surface of the crown and the demarcations established on its margins standardized its insertion in the steel die and in the stone dies, under constant load of 250 g. The occlusal surface of the metallic crown and the steel die were leveled at zero, which was considered the referential for the measurements obtained in the measuring microscope. 

**Figure 3:**
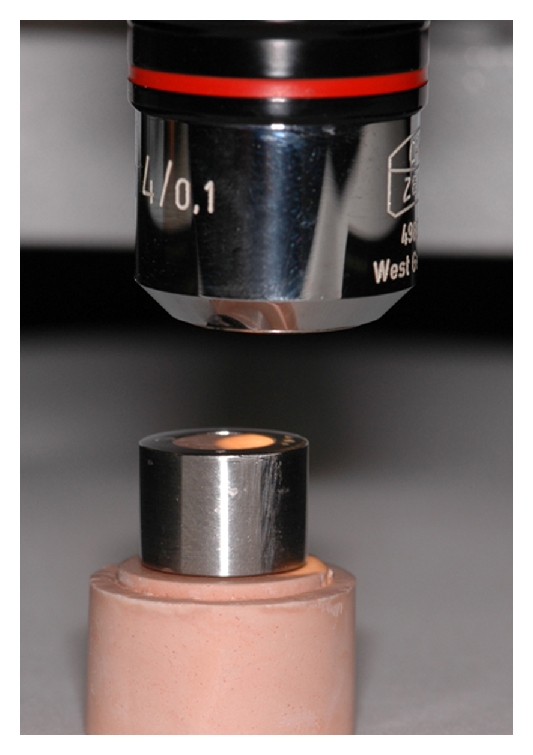
Metallic crown and die perpendicularly positioned under to the objective of the depth measuring microscope (160x magnification). The difference in height between the upper surface of the stone die and the crown was registered in micrometers.

The metallic crown and die were perpendicularly positioned under to the objective of the depth measuring microscope at 160x magnification (Figure 3). First, focus was determined on the occlusal surface of the metallic crown, and the microscope was set at zero. Then focus was determined at the occlusal surface of the stone die. The difference in height between the upper surface of the stone die and the crown was registered in micrometers. Four measurements were performed on each sample at demarcated points, by 3 independent examiners, and a mean value was calculated for each specimen. Data were submitted to the two-way analysis of variance and Tukey multiple comparison test (*α* = 0.05).

## 3. Results

The single-step technique resulted in positive discrepancies for both polyether and vinyl polysiloxane, which indicate that the dies were slightly larger than the steel die. The 2-step technique, on the other hand, produced negative discrepancies for both materials, which revealed that smaller dies resulted from this procedure. Significant differences were detected for impression materials (*F*_df1;39_ = 17.15; *P* = 0.0001), techniques (*F*_df1;39_ = 629.16; *P* = 0.0001), and the interaction effect (*F*_df1;39_ = 8.34; *P* = 0.0065), thus rejecting the null hypotheses. Tukey multiple comparison test (*α* = 0.05) was used to identify the significant differences among the tested groups (Table 1).

Stone dies obtained from 2-step polyether impressions were significantly smaller when compared to dies obtained from 2-step vinyl polysiloxane impressions (Impregum 2-step: −290.94 ± 71.64 *μ*m; Perfectim 2-step: −201.86 ± 28.58 *μ*m). Homogeneous grouping detected no significant differences in dies obtained from either polyether or vinyl polysiloxane with the single-step technique (Impregum single-step: 63.52 ± 16.60 *μ*m; Perfectim single-step: 79.40 ± 14.11 *μ*m). 

## 4. Discussion

The stress generated during the impression is an inherent characteristic of these materials. Several factors affect the accuracy of impressions, such as the direction of the setting contraction, the elastic recovery of the material, the evaporation of volatile components, or continued polymerization after removal of the impression. All these factors may influence the outcome of research studies, producing data variability. Although high-standard deviation was observed in all groups, data variability in studies of impression materials has been previously described [4, 16] and highlights the possibility of even greater discrepancies in clinical procedures. Variability may have resulted from the variation in the measurement technique or the real differences from the master model in the resulting dies, due to expansion of the gypsum, distortion of the impression, or shrinkage of the impression material [4].

The impression material had a significant effect for the 2-step technique in this study, likewise observed by Hung et al. [5] and Boulton et al. [2]. It is possible that the high hardness of the vinyl polysiloxane leads to 30% lower discrepancy of the 2-step technique (with vinyl polysiloxane) when the same technique was performed with polyether. Yet, no different outcome was produced by the type of material for the single-step impressions. It seems that the improvement of impression materials has reached such an extent that the precision of the impressions may be controlled more by the technique than the material itself [1, 13]. In fact, this study supports that the impression technique is relevant to the accuracy of dies. 

When considering the impression technique itself, single-step impressions obtained in this study resulted in slightly larger dies, which was also observed in previous studies [3, 6, 12]. The discrepancies detected are probably the result of incomplete elastic recovery of the polyether and vinyl polysiloxane [1]. 

Indeed, most significant strain may be expected when relining is performed without relief of the preliminary impression or the use of die spacers, which is the case of the hydraulic and hydrophobic technique. During reseating of the tray, the wash induces tension on the high-viscosity material, thus inducing deformation on the already set impression. After setting and on removal, the high-consistency material is likely to exhibit elastic recovery, returning to its original position [2, 5, 6, 16], thus resulting in smaller dies. This was observed in this study and the one conducted by Petersen and Asmussen [9]. Although the elevated hardness of the high-viscosity material indicates little flexibility and high degree of rigidity, which are desirable characteristics for an impression material, it was not capable to avoid the dimensional alteration of the vinyl polysiloxane in the 2-step technique without relief. Additionally, if no relief is performed on the preliminary impression, there is no space to allow the wash material to flow, which complicates the reset of the primary impression. 

The type of discrepancy may result in different clinical situations. Slight positive discrepancies, such as those observed in the single-step technique, are acceptable and may be advantageous in some situations, when larger dies may compensate the contraction developed during metal casting. Casts of the exact dimension of the preparation may make the adaptation more difficult and, consequently, establish marginal discrepancies. A small positive error may be desirable when making a full crown, because the cast would be slightly larger, and the crown is more likely to fit. Smaller dies, on the other hand, could be beneficial when fabricating intracoronal restorations, when a smaller cast is desired [4]. However, high-negative discrepancies, such as those detected in this study at the 2-step impressions without relief, may be clinically disadvantageous because a thick cement line is expected. Consequently, problems like higher cement solubility or degradation, marginal leakage, and secondary caries are likely to occur.

Different alternatives have been proposed to minimize the discrepancies resulting from impression taking. Relief of the preliminary impression, use of die spacers, and the use of a plastic sheet over the preliminary impression may be good alternatives to produce adequate space for the wash material to flow in the 2-step technique. In fact, minimal dimensional changes have been observed in stone dies when relief is performed in the preliminary impression for the 2-step technique, with similar results to dies acquired from single-step impressions [5–7, 11]. One alternative to obtain 1-2 mm of space for the low-consistency material, which is enough to acquire accurate stone dies, might be to utilize a temporary crown during the preliminary impression with the heavy-bodied material [3, 8].

Since impression taking is only one of the steps of a laboratory protocol, it is very likely that the final indirect restoration will not fit properly if impression inaccuracies are present. Larger misfit implies in thicker cement lines and all problems associated with it. Therefore, the selection of the appropriate impression technique is important in obtaining optimal results. 

It must be emphasized, however, that the data hereby expressed present the limitations of an *in vitro* study, and further clinical studies are desirable. This laboratory study investigated the accuracy of fit of a metallic crown on stone dies produced from a single preparation, under standardized conditions. In the clinical procedure, it is impossible to eliminate the effect of undercuts on the adjacent teeth and it is difficult to control exactly the thickness of the impression material around the preparation. Both of these factors are known to directly influence the accuracy of impressions [8, 15]. Therefore, this *in vitro* study offers guidelines for subsequent clinical research, which are imperative for confirmation of the laboratorial findings.

## 5. Conclusions

The 2-step hydraulic and hydrophobic impression technique resulted in significant discrepancies in stone dies when no relief of the preliminary impression was performed. The single-step technique produced smaller inaccuracies in the stone dies obtained from the impression materials investigated in this study, thus being advisable over the 2-step hydraulic and hydrophobic impression without relief. 

## Figures and Tables

**Table 1:** Discrepancies (Mean ± SD, in *μ*m) of stone dies obtained from the different impression materials and techniques. Homogeneous grouping determined by Tukey multiple comparison test (*α* = 0.05). Positive discrepancies indicate that the dies were larger, while negative discrepancies indicate that the dies were smaller, when compared to the original stainless steel die.

Group (*n* = 10)	Mean (*μ*m) ± SD	Homogeneous grouping*
Perfectim single-step	79.40 ± 14.11	a
Impregum single-step	63.52 ± 16.60	a
Perfectim 2-step	−201.86 ± 28.58	b
Impregum 2-step	−290.94 ± 71.64	c

*Mean values followed by different letters correspond to significant differences.

## References

[1] Craig RG (1988). Review of dental impression materials. *Advances in Dental Research*.

[2] Boulton JL, Gage JP, Vincent PF, Basford KE (1996). A laboratory study of dimensional changes for three elastomeric impression materials using custom and stock trays. *Australian Dental Journal*.

[3] Caputi S, Varvara G (2008). Dimensional accuracy of resultant casts made by a monophase, one-step and two-step, and a novel two-step putty/light-body impression technique: an in vitro study. *Journal of Prosthetic Dentistry*.

[4] Cayouette MJ, Burgess JO, Jones RE, Yuan CH (2003). Three-dimensional analysis of dual-arch impression trays. *Quintessence International*.

[5] Hung SH, Purk JH, Tira DE, Eick JD (1992). Accuracy of one-step versus two-step putty wash addition silicone impression technique. *Journal of Prosthetic Dentistry*.

[6] Johnson GH, Craig RG (1986). Accuracy of addition silicones as a function of technique. *Journal of Prosthetic Dentistry*.

[7] Nissan J, Laufer BZ, Brosh T, Assif D (2000). Accuracy of three polyvinyl siloxane putty-wash impression techniques. *Journal of Prosthetic Dentistry*.

[8] Nissan J, Gross M, Shifman A, Assif D (2002). Effect of wash bulk on the accuracy of polyvinyl siloxane putty-wash impressions. *Journal of Oral Rehabilitation*.

[9] Petersen GF, Asmussen E (1991). Distortion of impression materials used in the double-mix technique. *Scandinavian Journal of Dental Research*.

[10] Johnson GH, Mancl LA, Schwedhelm ER, Verhoef DR, Lepe X (2010). Clinical trial investigating success rates for polyether and vinyl polysiloxane impressions made with full-arch and dual-arch plastic trays. *Journal of Prosthetic Dentistry*.

[11] Idris B, Houston F, Claffey N (1995). Comparison of the dimensional accuracy of one- and two-step techniques with the use of putty/wash addition silicone impression materials. *Journal of Prosthetic Dentistry*.

[12] Takahashi H, Finger WJ (1994). Effects of the setting stage on the accuracy of double-mix impressions made with addition-curing silicone. *Journal of Prosthetic Dentistry*.

[13] Luthardt RG, Walter MH, Quaas S, Koch R, Rudolph H (2010). Comparison of the three-dimensional correctness of impression techniques randomized controlled trial. *Quintessence International*.

[14] Hoos JC (1996). A problem-solving impression technique. *Dentistry Today*.

[15] de Araujo PA, Jørgensen KD (1985). Effect of material bulk and undercuts on the accuracy of impression materials. *Journal of Prosthetic Dentistry*.

[16] Fusayama T, Iwaku M, Daito K, Kurosaki N, Takatsu T (1974). Accuracy of the laminated single impression technique with silicone materials. *Journal of Prosthetic Dentistry*.

